# Homozygous duplication identified by whole genome sequencing causes *LRBA* deficiency

**DOI:** 10.1038/s41525-021-00263-z

**Published:** 2021-11-18

**Authors:** Daniele Merico, Yehonatan Pasternak, Mehdi Zarrei, Edward J. Higginbotham, Bhooma Thiruvahindrapuram, Ori Scott, Jessica Willett-Pachul, Eyal Grunebaum, Julia Upton, Adelle Atkinson, Vy H. D. Kim, Elbay Aliyev, Khalid Fakhro, Stephen W. Scherer, Chaim M. Roifman

**Affiliations:** 1 The Centre for Applied Genomics (TCAG), Program in Genetics and Genome Biology, The Hospital for Sick Children, Toronto, M5G 0A4 ON Canada; 2 Deep Genomics Inc., Toronto, M5G 1M1 ON Canada; 3 Canadian Center for Primary Immunodeficiency and the Jeffrey Modell Research Laboratory for the Diagnosis of Primary Immunodeficiency, Toronto, M5G1X8 ON Canada; 4 Division of Immunology and Allergy, Department of Paediatrics, The Hospital for Sick Children, Toronto, M5G 1×8 ON Canada; 5 University of Toronto, Toronto, M5S 1A8 ON Canada; 6 Department of Human Genetics, Sidra Medicine, Doha, Qatar; 7 Department of Genetic Medicine, Weill-Cornell Medical College, Doha, Qatar; 8 Department of Molecular Genetics, University of Toronto, Toronto, M5S 1A8 ON Canada; 9 McLaughlin Centre, University of Toronto, Toronto, M5G 0A4 ON Canada

**Keywords:** Primary immunodeficiency disorders, Genetic testing

## Abstract

In more than one-third of primary immunodeficiency (PID) patients, extensive genetic analysis including whole-exome sequencing (WES) fails to identify the genetic defect. Whole-genome sequencing (WGS) is able to detect variants missed by other genomics platforms, enabling the molecular diagnosis of otherwise unresolved cases. Here, we report two siblings, offspring of consanguineous parents, who experienced similar severe events encompassing early onset of colitis, lymphoproliferation, and hypogammaglobulinemia, typical of lipopolysaccharide-responsive and beige-like anchor (*LRBA*) or cytotoxic T lymphocyte antigen 4 (*CTLA4*) deficiencies. Gene-panel sequencing, comparative genomic hybridization (CGH) array, and WES failed to reveal a genetic aberration in relevant genes. WGS of these patients detected a 12.3 kb homozygous tandem duplication that was absent in control cohorts and is predicted to disrupt the reading frame of the *LRBA* gene. The variant was validated by PCR and Sanger sequencing, demonstrating the presence of the junction between the reference and the tandem-duplicated sequence. Droplet digital PCR (ddPCR) further confirmed the copy number in the unaffected parents (CN = 3, heterozygous) and affected siblings (CN = 4, homozygous), confirming the expected segregation pattern. In cases of suspected inherited immunodeficiency, WGS may reveal a mutation when other methods such as microarray and WES analysis failed to detect an aberration.

## Introduction

The *LRBA* gene encodes the lipopolysaccharide-responsive and beige-like anchor (LRBA) protein, which is highly conserved across species and widely expressed in human tissues^1,2^. Mutations in the *LRBA* gene cause an immunodeficiency encompassing autoimmune and lymphoproliferative features as well as antibody deficiency^2,3^. Commonly, these patients present in infancy or childhood with colitis, lymphadenopathy, and recurrent infections^4^. Most mutations described so far are localized throughout the gene and no correlation was found to the clinical presentation^4^. While most mutations resulted in a complete loss of LRBA protein, some had residual expression^5^. LRBA colocalizes with cytotoxic T lymphocyte antigen 4 (CTLA4) in endosomal vesicles and appears to control its turnover^5^.

CTLA4 is expressed on activated and regulatory T cells^6^ and provides an inhibitory proliferative signal by competing with the T cell co-stimulatory receptor CD28^7^. CTLA4 molecules are recycled by trafficking from the membrane to the cytoplasm, and back, through activation cycles^8^. Mutations in *LRBA* result in reduced CTLA4 expression, thus allowing for unchecked immune dysregulation, providing a plausible explanation for the autoimmune/lymphoproliferative nature of the disorder. Indeed, mutations in *CTLA4* result in a similar clinical spectrum to *LRBA* deficiency^9^. Moreover, patients with *LRBA* deficiency improve clinically when treated with the CTLA4–immunoglobulin fusion drug abatacept^10^.

In a large published cohort with suspected *LRBA* deficiency, genetic analysis of the *LRBA* gene including whole-exome sequencing (WES) failed to show a mutation in a significant number of patients^4^, suggesting this technique may fall short on identifying some genetic aberrations. We report here a similar case where whole-genome sequencing (WGS) was used in an attempt to define the diagnosis of *LRBA* deficiency, while gene-panel sequencing, comparative genomic hybridization (CGH) array, and WES failed to do so.

WGS can be effectively used to detect copy number variants (CNVs) and other structural variants that would be missed by exome sequencing or by genotyping or genome hybridization arrays^11,12^, offering a tremendous opportunity to identify a molecular diagnosis for otherwise unresolved cases. Specifically, a pipeline that combines different methods based on read depth (CNVnator^13^ and Estimation by Read Depth with Single-nucleotide variants (ERDS)^14^) was shown to be able to detect copy number gains and losses ranging from megabase to kilobase size range with high sensitivity and specificity^11^.

## Results

### Patients

Case 1 was born at term to consanguineous parents of Iraqi descent. Chronic watery diarrhea started at the age of 3 months and continued in spite of dietary changes, antibiotic therapy, and periodic management of *Clostridium difficile* when identified. An endoscopy performed at 18 months revealed complete villus atrophy, focal cryptitis, and chronic lamina propria inflammation. Symptoms improved transiently with corticosteroid treatment, only to resurface at 2 years of age. This time, cytomegalovirus (CMV) was detected in the gut requiring treatment with ganciclovir for 3 months. In parallel, he developed chronic thrombocytopenia and suffered repeated episodes of pneumonia leading to chronic interstitial lung disease. Since the age of 6 years he received intravenous immunoglobulin (IVIG) for hypogammaglobulinemia. At the age of 8 years he developed severe multiorgan serositis with pericardial and pleural effusions and ascites. In addition to vast effusions, imaging detected markedly enlarged lymph nodes in the neck and chest. Enteritis worsened and he eventually required parenteral nutrition or G-tube feeding. He subsequently developed protracted fever, neutropenia, and thrombocytopenia suggestive of hemophagocytic lymphohistiocytosis (HLH). The diagnosis was confirmed by the laboratory findings of elevated CD163 and IL-2, as well as hemophagocytosis identified in a bone marrow biopsy. CMV and pseudomonas were cultured from his blood samples. In spite of immunosuppressive treatment, HLH continued to deteriorate causing liver failure and ultimately leading to his death due to multiorgan failure. While the potential diagnosis of *LRBA* or *CTLA4* deficiency was raised, repeated attempts to reach a genetic diagnosis, including WES, have failed. With no definitive diagnosis, the family refrained from considering a hematopoietic stem cell therapy (HSCT).

Case 2 is the younger sibling of case 1. Like his older brother, he developed chronic diarrhea, thrombocytopenia, and marked generalized lymphadenopathy and splenomegaly by the age of 3 years. Endoscopy and colonoscopy performed at 7 years showed marked pan enteritis with flattened villi in the duodenum and colon, and diffuse inflammatory infiltrates. He too was diagnosed with hypogammaglobulinemia and received IVIG replacement but had consistent CMV and Epstein-Barr virus (EBV) viremia. He subsequently developed chronic liver disease and a low-grade HLH which gradually worsened at the age of 9 years. Following a partial response to treatment with sirolimus he received a HSCT from an unrelated donor, in the absence of a HLA-matched related donor. After myeloablative conditioning, he was fully engrafted but developed severe stage 4 graft versus host disease (GvHD) at 1 month post-transplant leading to hepatocellular damage and vanishing bile duct syndrome. He died at the age of 10 years due to severe gut and liver GvHD and uncontrolled gastrointestinal bleeding.

### Whole-genome sequencing variant identification

The two affected siblings, both male, underwent WGS on the Illumina HiSeqX platform, with PCR-free library preparation and 150 bp paired-end reads, resulting in an average genome coverage of 35x and with over 98% of reads aligning to the reference genome.

First, we prioritized substitutions and small insertion/deletion (indel) variants that were rare (frequency <5%) and that impacted exonic sequence directly, or that could impact it indirectly by altering splicing or other regulatory sequences^15^. This resulted in 15,536 and 15,839 variants in the two siblings, of which 7897 were shared. We then sorted rare variants into five groups based on mode of inheritance (homozygous, X-chromosome hemizygous, potentially compound heterozygous, autosomal dominant, and potentially dominant constrained genes) and flagged genes implicated in primary immunodeficiency (PID, 400 genes) or predicted to have potential implication in PID (2402 additional genes at a 80% recall cutoff). Focusing on variants present in both siblings and of high quality, we identified 10 homozygous variants, 4 X-chromosome hemizygous variants with allele frequency <0.0001, and 4 variants in two genes forming potential compound heterozygous sets with frequency product <0.0001. None of these variants impacted a PID gene or a gene predicted to be implicated in PID. We also identified 11 variants of high quality, absent from the Genome Aggregation Database (gnomAD), occurring in autosomal dominant genes, and present in both siblings; none occurred in a PID gene, and only one occurred in a gene predicted to be potentially implicated in PID, but closer inspection revealed a mismatched disorder (*RUNX2*, Cleidocranial or metaphyseal dysplasia). In addition, we identified 6 variants of high quality, absent from gnomAD, and present in both siblings occurring in constrained genes that could act as dominant, of which none was predicted to be implicated in PID (see Supplementary Data 1). Low-quality variants, variants with higher frequencies, and variants not shared by the two siblings were also inspected, but no candidate was found. In conclusion, no rare substitutions or small indel provided an explanation of the patients’ immune condition.

**Fig. 1:**
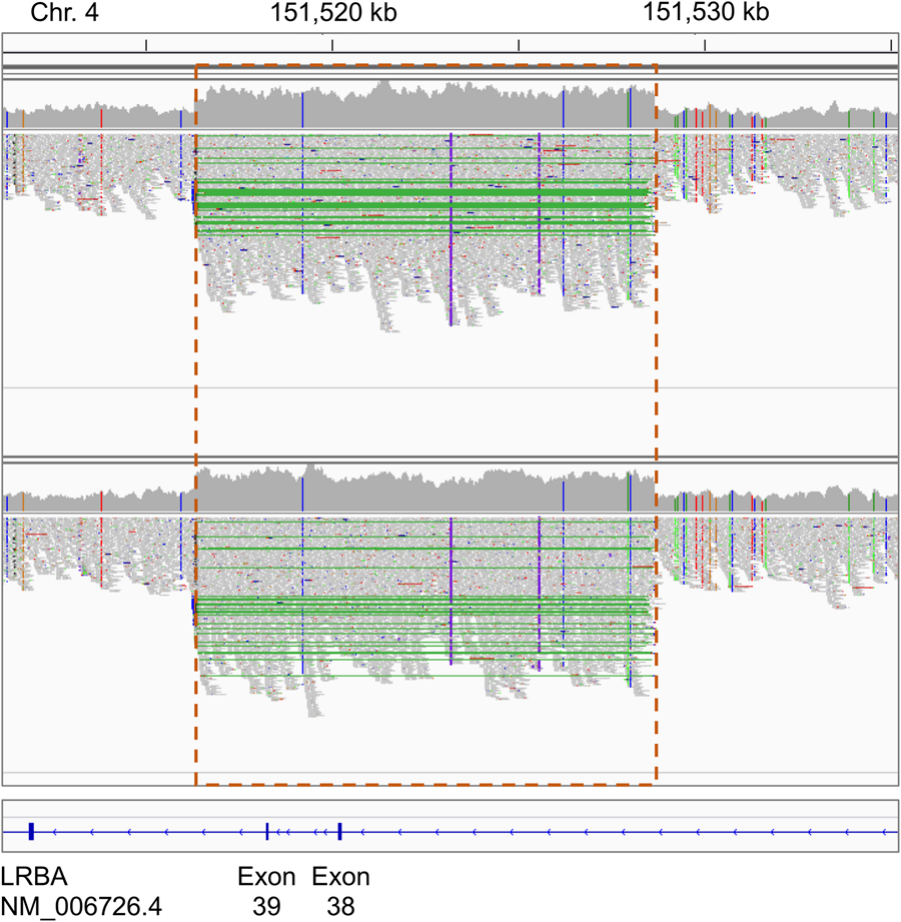
Read alignments at the copy number gain locus of chromosome 4. Read alignments for the two affected siblings were visualized using the Integrative Genomics Viewer (IGV)^45^. The orange dashed box indicates the duplicated region (copy number 4); green lines indicate paired-end reads spanning the junction between the original reference sequence and the tandem-duplicated sequence; vertical colored lines within the read pile-ups indicate substitutions (note that substitutions supported by multiple reads are all homozygous).

CNVs of size ≥1 kb were detected using WGS read depth^11^ and prioritized based on frequency, gene impact, and gene annotations. The two siblings shared only one rare variant impacting the exonic sequence of a gene implicated in PID or predicted to be potentially implicated in PID. In both siblings, the variant was detected on chromosome 4, with start position 151,516,001 and end position 151,529,000 (hg19/b37 coordinates) and was estimated to have copy number 4 (see Supplementary Data 2). This duplication was not observed in over 2500 unrelated parents of autism probands, sequenced by an Illumina platform and with CNV calls based on same read depth pipeline^16^, or in the Database of Genomics Variants (DGV) gold-standard dataset (23,300 subjects of various ethnicities)^17^, whereas Genome Aggregation Database - Structural Variants (gnomAD-SV) v2.1 (14,891 subjects)^12^ includes only a larger (131 kb) and ultra-rare duplication overlapping this locus (allele frequency ~0.00005). We additionally assessed the occurrence of any structural variation disrupting the LRBA gene in an internal database of 6941 genomes of predominantly Arab/Middle Eastern ethnicities, sequenced to a minimum of 30x depth^18^, but could not detect any. Inspection of the read alignments (BAM file) suggested more accurate coordinates for the duplication, demarcated by sharp transition of read depth, and that the duplication occurs in tandem (see Fig. 1). A 3 bp microhomology (ACT) was present at the start and at the end of the duplicated sequence, suggesting a mechanistic explanation for the duplication;^19^ Sanger sequencing later revealed that the microhomology is included only at the start but not at the end of the duplication, and thus the correct duplication coordinates are chr4:151,516,307-151,528,645 (length 12,339 bp; see next section for details). Since the duplication overlaps exons 38–39 (of 58 total exons) and surrounding intronic sequence of the *LRBA* transcript NM_006726.4 (which is predicted to be the principal transcript by APPRIS^20^), and since exon 39 is in-frame (length 33 bp) whereas exon 38 is out-of-frame (length 125 bp), we expect this tandem duplication to shift the *LRBA* reading frame and result in complete loss of function. It is noteworthy that exon 39 is not present in the Ensembl transcript ENST00000651943 (also predicted to be principal by APPRIS), and that GTEx junctional counts suggest a very low inclusion percentage (see Supplementary Fig. 1). However, the inclusion or exclusion of exon 39 does not alter the frameshift effect. In addition, the presence of >300 bp intronic sequence around the exons suggests that they will be spliced correctly in the tandem-duplicated region^21^. Finally, we observed that the ±10 kb region around the duplicated sequence is homozygous in both siblings (97–100% homozygous variants), in contrast to the overall rates for chromosome 4 (40–43%). This suggested that both the maternal and paternal allele present a tandem duplication and that both copies of *LRBA* are disrupted, which is consistent with the autosomal recessive mode of inheritance reported for *LRBA* and the disorder “Immunodeficiency, common variable, 8, with autoimmunity” (OMIM ID 614700). In conclusion, this *LRBA* multi-exonic duplication was considered a very compelling candidate to explain the immune disorder in the two siblings. We then proceeded to experimentally validate this variant and to confirm its correct segregation in the family.

### Variant validation and family segregation analysis

**Fig. 2:**
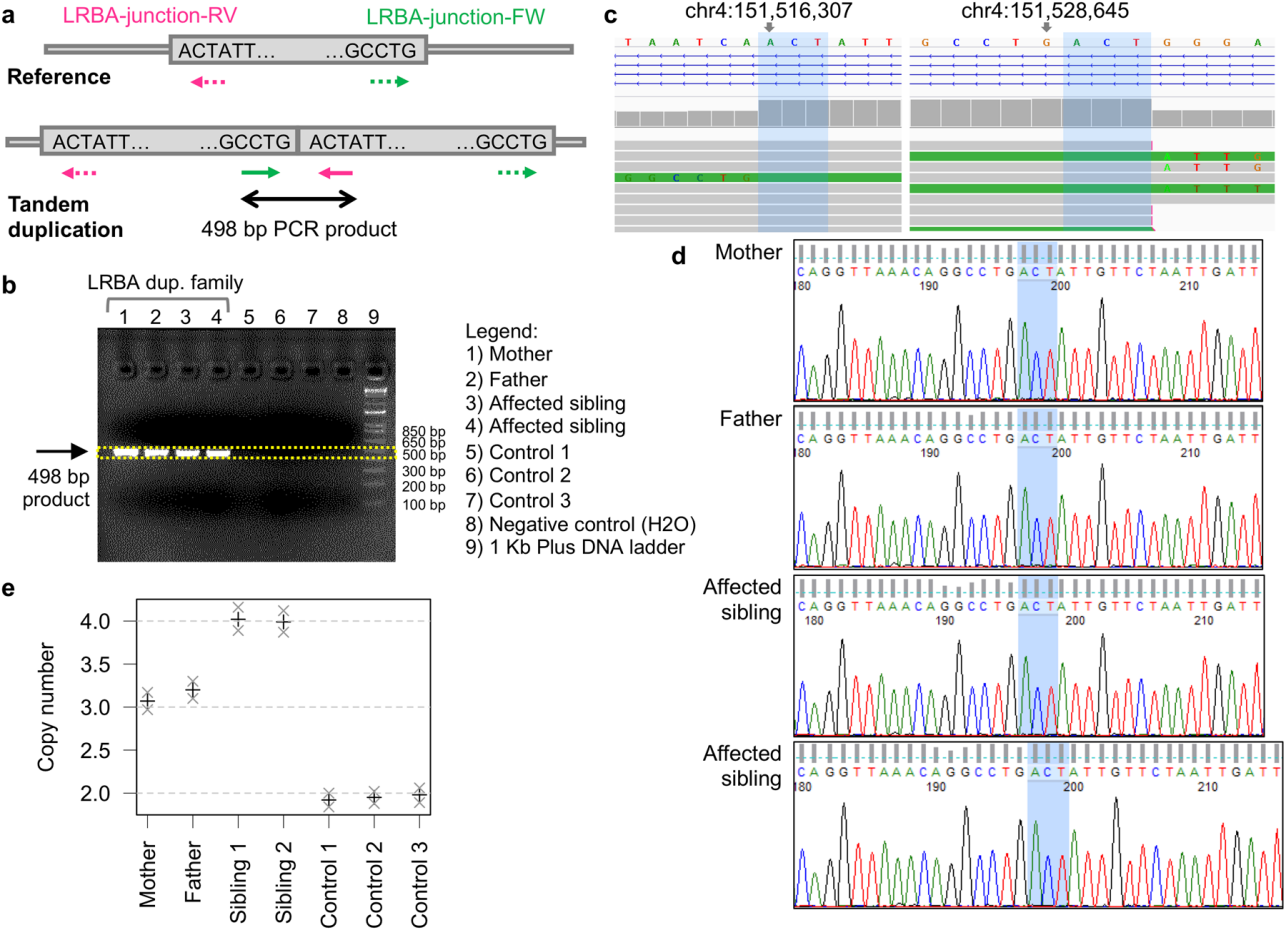
Validation and segregation experiments. **a** PCR primer design and breakpoint sequence (note that the sequence is not in scale relative to primer position). **b** PCR results, with the product corresponding to the tandem duplication junction present only in the four family members but not in controls. **c** Duplication breakpoints from WGS read alignment (visualized in IGV), note that this incorrectly suggests that the ACT microhomology sequence (blue shade) is present both at the beginning and the end of the duplication; also note that aligned reads displaying multiple substitutions (colored nucleotides within the read bars) correspond to junction-spanning reads and in fact present exactly the same sequence as identified by Sanger sequencing the junction PCR product. **d** Sanger sequencing of the PCR product showing the expected sequence. **e** ddPCR showing copy number 3 in the parents, copy number 4 in the affected siblings and copy number 2 in controls (+ indicates the point estimate, x indicates the 95% Poisson confidence interval).

We performed several experiments to demonstrate the presence of the tandem duplication and its correct segregation in the family. First, we performed PCR to demonstrate the presence of the junction between the reference sequence and the tandem-duplicated sequence in the two affected siblings and in their parents. Primers were designed to generate a product only in the presence of the tandem duplication (see Fig. 2a); an amplification product of the expected size was present in all four family members but not in unrelated controls (see Fig. 2b).

Then, we confirmed the sequence of the PCR-amplified junction by Sanger sequencing. Whereas WGS read alignments suggested that the duplicated sequence spans position 151,516,307–151,528,648 and includes the ACT microhomology sequence at both ends, Sanger sequencing of the junction showed the presence of the ACT sequence only once (see Fig. 2d), thus the correct coordinates are chr4:151,516,307–151,528,645. This discrepancy is the consequence of WGS reads being aligned to the reference sequence as opposed to the alternate sequence with the tandem duplication; therefore, reads spanning the tandem duplication junction extend into the ACT sequence after the end of the duplicated sequence, rather than being split-aligned to the beginning of the duplicated sequence where they belong (see Fig. 2c). Finally, we performed droplet digital PCR (ddPCR) with the TaqMan copy number assay to demonstrate that the duplication locus has copy number 2 in control samples, copy number 3 in the parents, and copy number 4 in the affected sibling (see Fig. 2e). In combination with the PCR and Sanger results, this conclusively proves that the tandem duplication alters both *LRBA* alleles in the affected siblings, but not in the parents.

### Detectability by other genomics platforms

Based on probe coverage reported by the DGV^17^, this duplication may be detected by Affymetrix CytoScan HD, but not by other single-nucleotide polymorphisms (SNP) or CGH array platforms (Agilent 244k, Affymetrix SNP Array 5.0, Affymetrix SNP Array 6.0, Illumina HumanHap 300, Illumina HumanHap 550, Illumina 610 Quad, Illumina HumanHap 650Y, Illumina Human 660 W, Illumina HumanHap 1 M) (see Supplementary Fig. 2). It is also noteworthy that, if detected by Cytoscan HD, it would pass previously established “research-grade” but not “clinical-grade” quality thresholds^22^. Reliable detection of CNVs from WES can be accomplished when CNVs span at least three exons^23,24^, thus this duplication would not be detectable. In addition, for both WES and array platforms, follow-up experiment would be required to determine its tandem configuration, whereas this is readily evident from WGS read alignment.

## Discussion

Mutations in the *LRBA* gene located on 4q31.3 and encoding the LRBA proteins are associated with an autosomal recessive immunodeficiency (OMIM#614700). The hallmarks of this deficiency consist of hypogammaglobulinemia, lymphoproliferation, and autoimmunity. Patients frequently present early in infancy with recurrent infections, lymphadenopathy, and enlarged spleen and liver, as well as a variety of autoimmune features including inflammatory bowel disease, autoimmune cytopenias, diabetes, and autoimmune hepatitis^2^.

LRBA, a cytoplasmic protein, interacts with CTLA4, and defects in LRBA interfere with its expression, thus mimicking *CTLA4* deficiency^9^. Indeed, the two conditions share clinical manifestations and respond well to treatment with abatacept, the CTLA4–immunoglobulin fusion drug^10^.

The gold standard of diagnosing *LRBA* deficiency is demonstrating a genetic aberration. Indeed, definitive diagnosis in a large cohort of phenotypical *LRBA* deficiency was attained by demonstrating biallelic mutations in the *LRBA* gene^4^. Deleterious mutations were located throughout the whole gene and most variants were missense, indels, splice site, or nonsense mutations^3^ with rare cases of uniparental isodisomy^25^.

Evaluation of LRBA and CTLA4 protein expression may aid in the evaluation, but are themselves insufficient to establish a diagnosis^26^. Clinical tests are not widely available, and frequently the results are inconclusive, limiting their use in clinical practice.

We report here two siblings who had a similar disease course which was typical of previously reported cases of *LRBA*/*CTLA4* deficiency. They had early onset colitis, recurrent infections associated with hypogammaglobulinemia, and enlarged liver, spleen and lymph nodes. In spite of this classic *LRBA* deficiency-associated phenotype, Sanger sequencing and WES failed to identify a mutation in either the *LRBA* or *CTLA4* gene.

We have subsequently performed WGS that revealed a novel 12.3 kb length tandem duplication on chromosome 4 impacting the exonic sequence of the *LRBA* gene. Both brothers were homozygous for this variant while parents carried the change only on one allele. This duplication overlaps exons 38–39 and surrounding intronic sequence of the *LRBA* principal transcript. Since exon 39 is in-frame whereas exon 38 is out-of-frame, this tandem duplication was expected to shift the *LRBA* reading frame, resulting in loss of function. Moreover, the region adjacent to the duplicated sequence was homozygous in both siblings suggesting both maternal and paternal copies are equally disrupted, which is expected in an autosomal recessive condition.

We then went on to validate the variant by demonstrating the presence of the junction between reference sequence and the tandem duplication. This was confirmed by Sanger sequencing of the PCR-amplified junction. Copy number was then examined by performing ddPCR, verifying that while 2 copies existed in wild-type controls, 3 and 4 copies were deleted in parents and patients, respectively. Together, these experiments show conclusively that the tandem duplication alters both alleles in the affected siblings and correctly segregates in the family.

Intragenic exon duplications often lead to loss or alteration of function, as suggested by the depletion observed in the gnomAD-SV dataset for this type of variants in genes under constraint for heterozygous protein-truncating variants. While this depletion is more modest than for copy number losses, the ultimate effect of intragenic exon duplication depends on the sequence context and presence or absence of frameshift^12^. Intragenic homozygous duplications have been previously reported to cause recessive disorders, although more rarely than other types of structural variants^27^. Intragenic duplications expected to shift the reading frame or to cause other profound reading frame alterations have been previously reported as (likely) pathogenic for immune^28–30^ and non-immune disorders^31,32^.

This specific duplication could not be found among individuals of European descent or among a large database of WGS individuals of Arab and Middle Eastern ancestries, and thus may be extremely rare or restricted to one kin.

These results explain why we were unable to detect the mutation by the CGH array platform, as deduced from DGV probe coverage, nor by WES, which is able to detect larger CNV’s spanning at least 3 exons.

Together, the typical clinical features coupled with the convincing genomic analysis provided a compelling argument for the identified duplication causing *LRBA* deficiency in these patients. The diagnosis of *LRBA* deficiency is critically important in order to chart a course of treatment. Both innovative CTLA4–immunoglobulin construct as well as curative HSCT are effective in preventing severe outcome if applied early. In this report we have shown that the delay in diagnosis due to failure to detect a pathogenic LRBA variant by WES and Sanger sequencing likely contributed to the demise of the patients. This clearly highlights the need to make WGS a clinically accessible test. Identifying the mutation by WGS aided in genetic counseling as well as treatment planning for future offspring in the affected family.

## Methods

### Patients

All patient data and samples were obtained in accordance with the Research Ethics Board at The Hospital for Sick Children. Patient data was compiled from medical records and entered into the Primary Immunodeficiency Registry and Tissue Bank (REB protocol no. 1000005598). Written informed consent was obtained from all participants for genetic testing, including WGS.

### Whole-genome sequencing

First, 6 μg of genomic DNA were submitted to TCAG (Toronto, Canada) for genomic library preparation and WGS. TCAG quantified DNA samples using Qubit High Sensitivity Assay and checked sample purity using Nanodrop OD260/280 ratio. Then, 700 ng of DNA was used as input material for library preparation using the Illumina TruSeq PCR-free DNA Library Prep Kit following the manufacturer’s recommended protocol. In brief, DNA was fragmented to 400 bp on average using sonication on a Covaris LE220 instrument; fragmented DNA was then end-repaired, A-tailed, and indexed TruSeq Illumina adapters with overhang-T were added to the DNA; libraries were validated on a Fragment Analyzer Using High Sensitivity NGS Kit to check for size and absence of primer dimers, and quantified by qPCR using Kapa Library Quantification Illumina/ABI Prism Kit protocol (KAPA Biosystems). Validated libraries were pooled in equimolar quantities and paired-end sequenced on an Illumina HiSeq X platform following Illumina’s recommended protocol to generate paired-end reads of 150 bases in length.

### Whole-genome read alignment and variant calling

Base calling was performed using the HiSeq Analysis Software. Reads were mapped to the b37 reference sequence using the BWA-MEM algorithm^33^. Duplicate reads were marked using Picard Tools. Local realignment and base quality score recalibration were performed using GATK 3.7^34^. Variants were called using HaplotypeCaller (GATK 3.7).

CNVs, comprising losses and gains with size ≥1 kb, were called using a pipeline based on the read depth callers ERDS and CNVnator^11^. Gains with size 1–5 kb supported only by ERDS were included.

### Variant annotation and prioritization

For substitutions and small indels, variants were defined as 5% rare when they had allele frequency ≤5% in all gnomAD 2.1.1 ethnic populations^35^. High-quality variants were defined as having GATK filter PASS, DP ≥ 6 and not overlapping a segmental duplication. In addition, heterozygous single-nucleotide substitutions were required to have FisherStrand ≤ 60.0, mapping quality ≥ 40.0, MQRankSum ≥ −12.5, and ReadPosRankSum ≥ −8.0; homozygous single-nucleotide substitutions were required to have FisherStrand ≤ 60.0 and mapping quality ≥ 40.0; heterozygous indels were required to have FisherStrand ≤ 200 and ReadPosRankSum ≥ −20; homozygous indels were required to have FisherStrand ≤ 200. Unless explicitly indicated, all variants called by GATK haplotype caller were used. Annovar (April 2018 version)^36^ was used to determine the variant effect on coding and non-coding genes, using the RefSeq database (Annovar refGene database, downloaded August 2019). We initially selected 5% rare variants impacting coding exons, non-coding exons, 5′ or 3′ UTRs, regions 1 kb upstream of transcription starts site or 1 kb downstream of transcription end site, variants within 100 bp of a splice site or predicted to alter splicing by Spidex (absolute dPSI > 2)^21^, dbscSNV (ADA or RF score > 0.6)^37^, or SpliceAI^38^. When considering specific modes of inheritance, coding and splicing variants were further prioritized based on their impact scores^39^. Constrained genes were defined as having gnomAD observed/expected LOF variants < 0.25 or observed/expected missense variants < 0.7 (where LOF indicates variants predicted to result in complete loss of function)^35^.

For CNVs, high-quality CNVs^11^, including gains with size 1–5 kb supported only by ERDS, were deemed rare if they had frequency <1% with respect to CNVs with >50% reciprocal overlap detected in parents of individuals with autism spectrum disorder in the MSSNG whole-genome sequencing project and sequenced by HiSeq 2000 or HiSeq X^16^.

Known PID were derived from the Genomics England (GE) primary immunodeficiency panel v2.368 (https://panelapp.genomicsengland.co.uk/panels/398/). Potential implication in PID was predicted using a generalized boosted regression model. The model was trained using the R package *gbm* version 2.1.8 and 4-fold cross-validation. The PID GE panel was used as known labels for supervised classification, and the features for prediction were constructed from the Human Protein Atlas RNA expression consensus dataset^40^, immune-related Gene Ontology annotations^41^ and pathways (KEGG^42^, Reactome^43^) and MGI immunity-related phenotypes^44^. At 80% recall, 2402 non-PID genes were predicted to be potentially implicated in PID.

APPRIS 2020_06.v32 was used to determine principal transcripts^20^.

We used the 2016-05-15 release of the DGV gold-standard, which includes 10,451 subjects of undetermined ethnicity, 9022 European, 1378 African, 1030 East Asian, 569 South Asian, 339 Latin American, 178 Middle Eastern, and 333 of other ethnicities.

### Variant validation and segregation

The following primers were designed to amplify the sequence spanning the tandem duplication junction: LRBA-Junction-FW primer sequence, ACACGGCAGCAACATACA (hg19/b37 coordinates chr4:151528419-151528436); LRBA-Junction-RV primer sequence, CTAGGGATGACAGATCATGTAAAG (hg19/b37 coordinates chr4:151516554-151516577). The PCR reaction was run using 20 ng of genomic DNA per sample in a 20 μl PCR reaction, with Qiagen HotStarTaq polymerase. Samples were run for 15 min at 95 °C (initial denaturation); followed by 36 cycles of (1) 30 s at 95 °C (denaturation), (2) 30 s at 60 °C (annealing), (3) 1 min at 70 °C (extension); followed by 10 min at 70 °C (final extension). The PCR product was visualized on a 2% agarose gel. All gels were derived from the same experiment and were processed in parallel.

ddPCR with TaqMan assays was used to determine copy number within the duplication in the four family members, and specifically using the probe Hs00518898_cn (hg19/b37 probe coordinate chr4:151520163, overlaps exon 38, https://www.thermofisher.com/order/genome-database/details/cnv/Hs00518898_cn). The TaqMan copy number reference assay based on human RNase P was used as an endogenous control for calibration. The assay was performed with one biological replicate per subject and results were analyzed using QuantaSoft Version 1.7.4.

### Reporting summary

Further information on research design is available in the Nature Research Reporting Summary linked to this article.

## Supplementary information

Supplementary information

Supplementary Data 1

Supplementary Data 2

Reporting summary

## Data Availability

Whole-genome sequencing data that support the findings of this study have been deposited in dbGaP with the accession code “phs002557”.
