# Supplementary material for: Homozygous duplication identified by whole genome sequencing causes LRBA deficiency
Source: NPJ Genom Med. 2021 Nov 18;6:96. doi: 10.1038/s41525-021-00263-z (PMC8602677; doi:10.1038/s41525-021-00263-z)
Supplement: Supplementary file 1 — Supplementary information [file 41525_2021_263_MOESM1_ESM.pdf]

SUPPLEMENTARY DATA

Homozygous duplication identified by whole genome sequencing causes *LRBA* deficiency

Daniele Merico[a,b], Yehonatan Pasternak[d,e,f], Mehdi Zarrei[a], Edward J. Higginbotham[a], Bhooma Thiruvahindrapuram[a], Ori Scott[d,e,f], Jessica Willett-Pachul[e], Eyal Grunebaum[e,f], Julia Upton[e,f], Adelle Atkinson[e,f], Vy H.D. Kim[d], Elbay Aliyev[g], Khalid Fakhro[f,h], Stephen W. Scherer[a,c], Chaim M. Roifman[d,e, f]*

* corresponding author

a - The Centre for Applied Genomics (TCAG), Program in Genetics and Genome Biology, The Hospital for Sick Children, Toronto, M5G 0A4, Ontario, Canada
b - Deep Genomics Inc., Toronto, M5G 1M1, Ontario, Canada
c - Department of Molecular Genetics, University of Toronto, Toronto, M5S 1A8, Ontario, Canada; McLaughlin Centre, University of Toronto, Toronto, M5G 0A4, Ontario, Canada
d - Canadian Center for Primary Immunodeficiency and the Jeffrey Modell Research Laboratory for the Diagnosis of Primary Immunodeficiency, Toronto, M5G1X8, Ontario, Canada
e - Division of Immunology and Allergy, Department of Paediatrics, The Hospital for Sick Children, Toronto, M5G 1X8, Ontario, Canada
f - University of Toronto, Toronto, M5S 1A8, Ontario, Canada
g - Department of Human Genetics, Sidra Medicine, Doha, Qatar
h - Department of Genetic Medicine, Weill-Cornell Medical College, Doha, Qatar

Address for Correspondence:
Dr. Chaim M. Roifman, CM, MD, FRCPC, FCACB. Hospital for Sick Children, University of Toronto, Toronto, Ontario, Canada
Email: chaim.roifman@sickkids.ca   Phone: 416-813-8629   Fax: 416-813-5932

FIGURE LEGENDS

Supplementary Figure 1: GTEx junction analysis showing that junction 29, skipping exon 39, is predominantly expressed in all tissues.

Supplementary Figure 2: Probes for different SNP and CGH array platforms in the duplicated genomic region, as provided by DGV.

Supplementary Data 1: Prioritized rare SNVs and indels from WGS.

Supplementary Data 2: Rare CNVs from WGS.

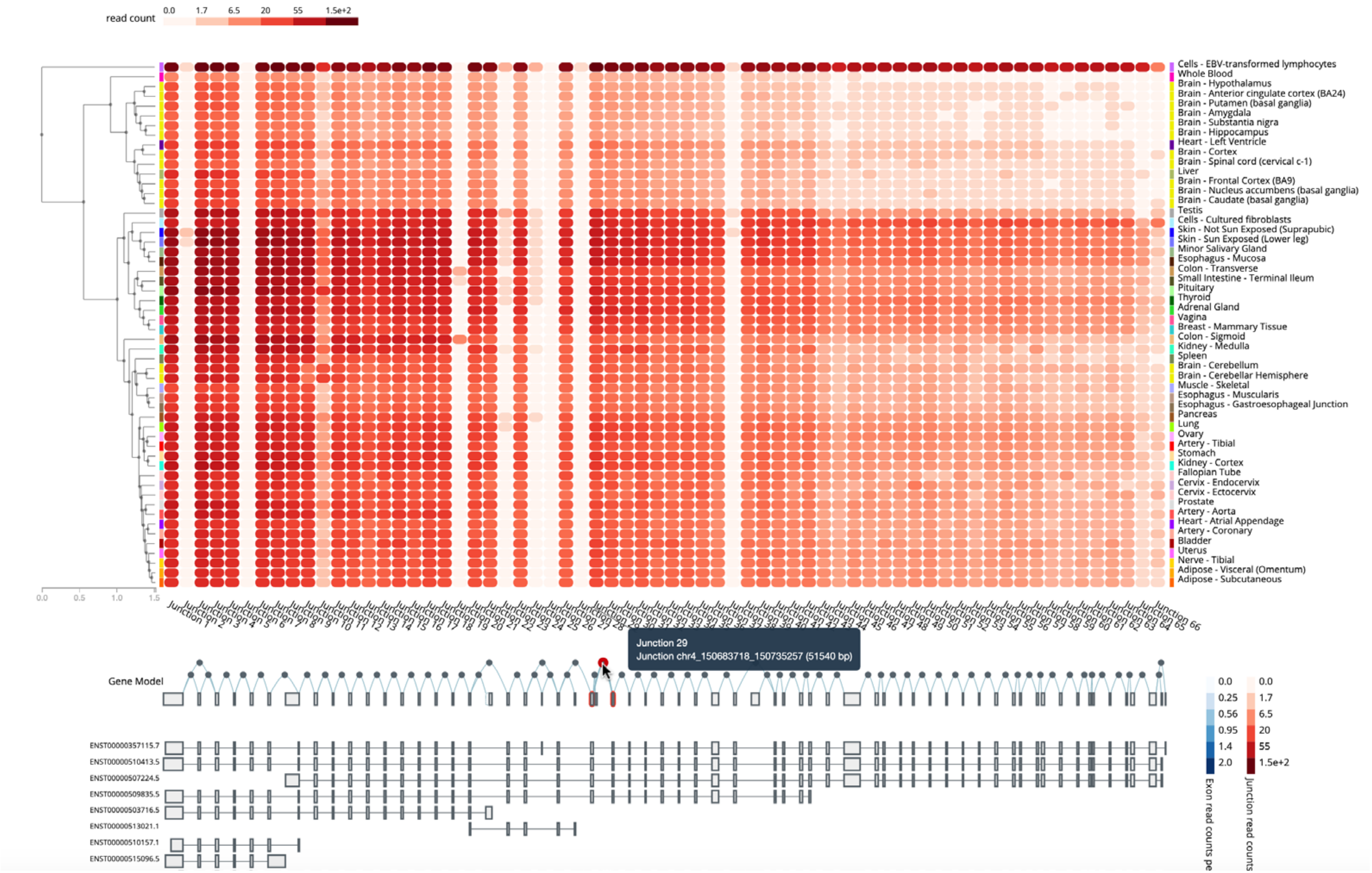

Supplementary Figure 1: GTEx junction analysis showing that junction 29, skipping exon 39, is predominantly expressed in all tissues.

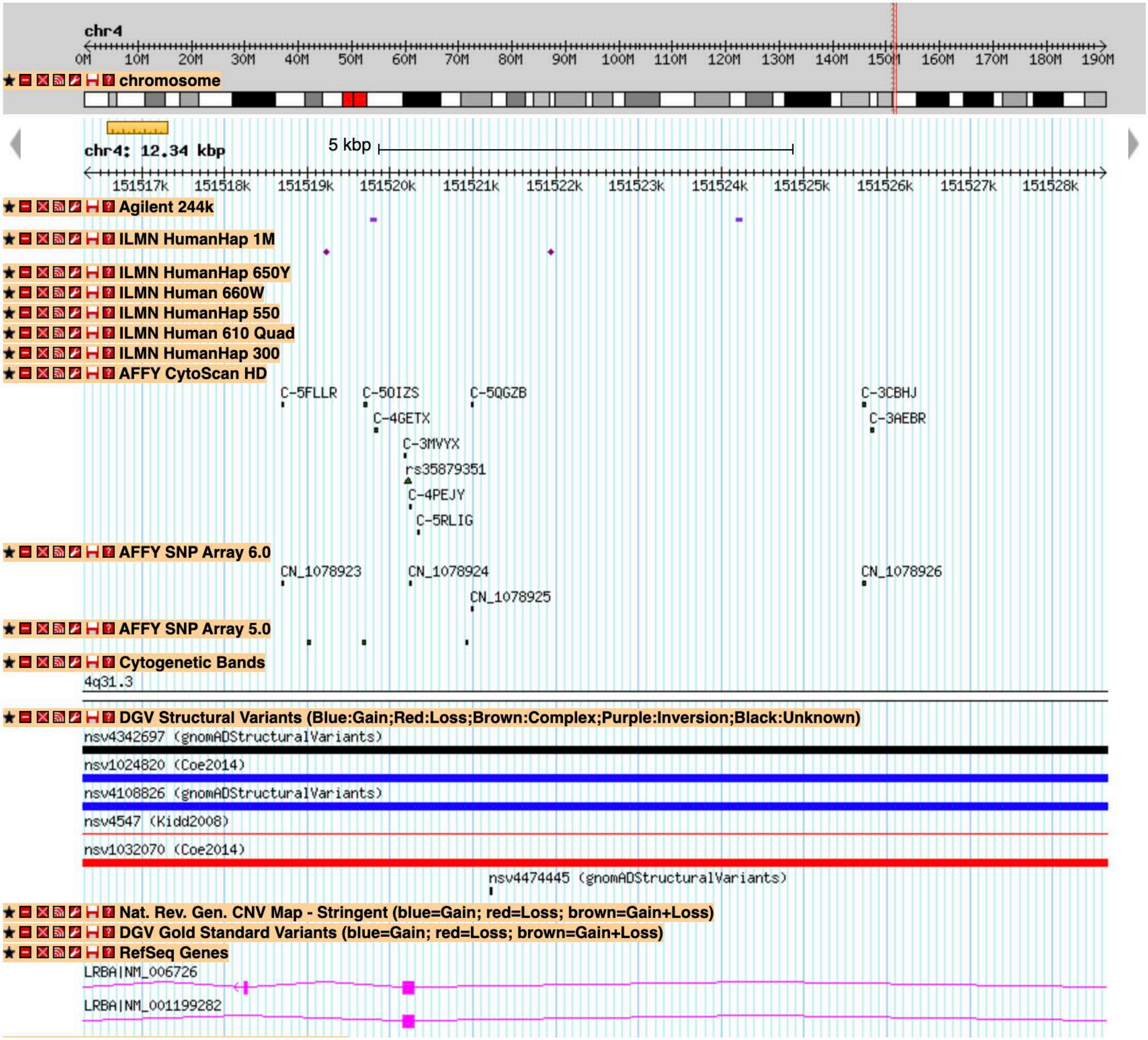

Supplementary Figure 2: Probes for different SNP and CGH array platforms in the duplicated genomic region, as provided by DGV.
